# Genomic Characterization and Probiotic Potency of *Bacillus* sp. DU-106, a Highly Effective Producer of L-Lactic Acid Isolated From Fermented Yogurt

**DOI:** 10.3389/fmicb.2018.02216

**Published:** 2018-09-20

**Authors:** Pan Li, Wenni Tian, Zhuo Jiang, Zuanhao Liang, Xueyin Wu, Bing Du

**Affiliations:** College of Food Science, South China Agricultural University, Guangzhou, China

**Keywords:** *Bacillus* sp. DU-106, complete genome sequence, lactic acid, probiotics, virulence gene

## Abstract

*Bacillus* sp. DU-106, a newly isolated member of *Bacillus cereus* group, exhibits the predominant ability to produce L-lactic acid. The probiotic potency of test strain revealed its survivability at acidic pH, bile salts and viability in simulated gastric juice *in vitro*. The acute oral toxicity test indicated its no toxicity to laboratory mice *in vivo*. We further determined the complete genome of strain DU-106 to understand genetic basis as a potential probiotic. It has a circular chromosome and three plasmids for a total genome 5,758,208 bp in size with a G + C content of 35.10%. Genes associated with lactate synthesis were found in the DU-106 genome. We also annotated various stress-related, bile salt resistance, and adhesion-related domains in this strain, which likely provide support in exerting probiotic action by enabling adhesion to host epithelial cells and survival under gastrointestinal tract. Moreover, strain DU-106 genome lacks the virulence genes encodes cereulide synthetase, enterotoxin FM, and cytotoxin K. These phenotypic and genomic probiotic potencies facilitate its potential candidate as probiotic starter in food industry.

## Introduction

Lactic acid has been widely used as a valuable chemical in food industries. Nowadays, lactic acid bacteria including *Lactobacillus rhamnosus* and *Lactococcus lactis* are frequently used in industrial production of lactic acid (Okano et al., 2010). Recently, some thermo-tolerant *Bacillus* members, including *Bacillus coagulans* (Zhang, 2014), *Bacillus licheniformis* (Wang et al., 2011), and other *Bacillus* strains (Wang et al., 2010; Poudel et al., 2015), were considered as new lactic acid producers. Compared to the lactic acid fermentation under mesophilic conditions, lactic acid producers of thermophilic *Bacillus* species have been shown to be promising in industrial-scale fermentations with a low energy and a low risk for contamination (Poudel et al., 2015).

Probiotics are live microorganisms that confer beneficial effects on the host (Hill et al., 2014). Most commonly used probiotic bacteria are autochthonous mainly including the lactic acid bacteria belonging to the genera of *Lactobacillus* and *Bifidobacterium*. Meanwhile, Hong et al. (2009) reported that *Bacillus subtilis* and probably other species are human gut commensals. *Bacillus* probiotics are currently of keen interest to the probiotic industry as they can be marketed in the spore form, which was easier to survive through the gastrointestinal tract and has indefinite shelf life (Hong et al., 2010). Recently, probiotic *Bacillus* species available in the market include *Bacillus subtilis*, *Bacillus cereus*, *Bacillus licheniformis*, *Bacillus pumilus*, *Bacillus clausii*, and *Bacillus coagulans* (Hong et al., 2010; Indu et al., 2016).

*Bacillus coagulans* is unique in that it shows characteristics of both Bacillaceae and Lactobacillaceae. It shares certain characteristics including the outstanding ability of lactic acid production (Juturu and Wu, 2017). It has been demonstrated to show a positive effect in the treatment of irritable bowel syndrome (Majeed et al., 2015), bacterial vaginosis (Ratna et al., 2012), and gingivitis (Alkaya et al., 2016) by several clinical trials. In this communication, we reported a *Bacillus* isolates with predominant ability to produce L-lactic acid and also evaluated its properties to use as a potential probiotic.

## Materials and Methods

### Isolation of DU-106 and Evaluation of Its L-Lactic Acid Production Capacity

*Bacillus* sp. DU-106 was isolated from traditional fermented yogurt with the De Man, Rogosa and Sharpe (MRS) broth (Difco, Paris, France) at 37°C. The strain was firstly activated at 37°C with MRS agar and was introduced to 5 mL MRS broth. Then it was incubated at 37°C for 24 h on a shaking incubator (Yuejin THZ-82A, Shanghai, China) at 150 rpm, which was used as the seed culture. The lactic acid fermentation was carried out in 500 mL conical flasks containing 200 mL MRS at 38°C and 150 rpm broth with an inoculation rate of 1% (v/v). The samples were taken every 12 h for further analysis.

The pH was measured by a digital pH Meter (Inesa pHs-3C, China). The L-lactic acid concentration was determined by High performance liquid chromatography (HPLC) (Shimadzu, LC-20AT, Japan) with a WondaSil C18-WR (4.6 × 250 mm, 5 μm) column at 35°C using 0.2% metaphosphoric acid solution as the elution at a flow rate of 0.8 mL/min, and quantified with a UV-VIS detector at 210 nm.

### Identification of DU-106 by 16S rRNA Sequencing

The genomic DNA of strain DU-106 was extracted using the Qiagen DNA extraction kit. The 16S rRNA genes was PCR-amplified according to previously reports (Garcia et al., 2016), and then sequenced by Invitrogen (Shanghai, China). The neighbor-joining phylogenetic tree was constructed with MEGA 6.0 software with a bootstrap value of 1,000 (Houfani et al., 2017). Gram staining was performed by using a Gram-stain reagent kit (HuanKai, Guangzhou, China).

### Potential Probiotic Characterization *in vitro*

For acid tolerance test, 6 × 10^7^ cfu/mL of strain DU-106 were dissolved in 0.1 M sodium citrate-hydrochloric acid buffer solution (pH 1.55, 2.42, 4.94). For bile tolerance, 6 × 10^7^ cfu/mL of strain DU-106 were inoculated in 0.01 M PBS buffer (pH 7.0) containing 0.3% bile salt. For simulated gastric and intestinal fluid tolerance, 6 × 10^7^ cfu/mL of strain DU-106 were inoculated in the artificial gastric juice (CZ0211, LEAGENE, Beijing, China) and artificial intestinal juice (CZ0201, LEAGENE, Beijing, China). The survival rates was calculated by measuring the survival cell counts after incubation at 37°C for 2 h using plate counting in MRS agar and expressed as percentage of the original cell counts.

### Toxicological Evaluation *in vivo* With Laboratory Mice

The *in vivo* toxicological evaluation of DU-106 was carried out in Guangdong Medical Laboratory Animal Center (Guangzhou, China) using the BALB/c and KM mice with certificate of conformity No. SCXK 2013-0002. Both of twenty BALB/c and KM healthy mice were randomly divided into two groups, each group consisted of five males and five females. The BALB/c mice were administered with the bacterial doses of 3 × 10^8^ cfu per mouse per day by continuous oral gavage for 7 days using a sterile pipette (Benjakul et al., 2018). For the acute oral toxicity test, the KM mice were orally administered with the initial bacterial doses of 1 × 10^12^ cfu Kg^−1^ body weight and then were observed for 14 days. The mice of the control group were administered using the same amount of physiological saline.

The body weight of each BALB/c and KM mouse was recorded on days 0 and 7, or recorded on days 0, 1, 3, 7, and 14. At the end of the experiment, all the surviving BALB/c mice were sacrificed by euthanasia, the D-lactic acid level of blood was determined by the D-lactic acid ELISA kit. Full gross pathological examination of the organs was conducted. The median lethal dose (LD_50_) was calculated according to previously (Yang et al., 2016). All animal experiments were performed in compliance with the principles of the Animal and Ethics Review Committee of Guangdong Medical Laboratory Animal Center, and Ethics Review Committee of South China Agricultural University approved the protocols used in this study.

### Complete Genome Sequencing and Annotation

Complete genome sequencing of strain DU-106 was carried out by a combined strategy of PacBio RSII sequencing and Illumina HiSeq 4000 sequencing technology. SOAPnuke and SMRT analysis 2.3.0 software were performed to filter the raw data (Chin et al., 2013). RS_HGAP Assembly3 in SMRT analysis v2.3.0 was employed to assemble the clean Pacbio subreads (Evivie et al., 2017). Then, the assembled fragments were further aligned and corrected with HiSeq clean data using SOAPaligner 2.21, and were further scaffolded and checked to produce a circular chromosome using SSPACE-LongRead (Khaleque et al., 2017). The protein coding sequences were predicted with Glimmer 3.02 (Delcher et al., 2007), and annotations of gene functions were performed according to NCBI Prokaryotic Genome Annotation Pipeline. The ncRNA was annotated with Rfam database, and was predicted with cmsearch program under default parameters by Rfam. The micro- and mini-satellite DNA were searched using the RepeatMasker v3-3-0 and Tandem Repeats Finder (TRF) software v4.04 (Saha et al., 2008).

### Comparative Genome Analysis

Six genomes of *Bacillus* strains, including *Bacillus cereus* ATCC 14579, *Bacillus thuringiensis* ATCC 10792, *Bacillus toyonensis* BCT-7112, *Bacillus mycoides* ATCC 6462, *Bacillus pseudomycoides* DSM 12442, and *Bacillus coagulans* ATCC 7050, were chosen for comparative genomics analysis with *Bacillus* sp. DU-106. The gene families were extracted by Hcluster-sg 0.5.1 with default parameters (Lopes et al., 2017). Orthologous genes between all organisms were detected with Proteinortho (Lechner et al., 2011) including protein blast with a similarity cut-off of (50%) and an *E*-value of 1e^−10^ (Hollensteiner et al., 2017). Multiple sequence alignment was performed using PRANK v140110 (Loytynoja and Goldman, 2005). Genomic synteny was analyzed on the basis of the results of the alignment, which was conducted using MUMmer v3.23 and LASTZ v1.03.54 tools between DU-1061 and referenced genome under default parameters (Tang et al., 2011; Chen et al., 2018). The potential positions of single nucleotide polymorphisms (SNPs) were primary generated by MUMmer v3.23, and were further identified by BLAT 35 (Kent, 2002). Finally, the SNPs from potential paralogous regions were excluded and confirmed with RepeatMasker and TRF (Saha et al., 2008). The maximum likelihood phylogenetic tree was constructed with PhyML 3.0 based on SNP differences across the whole genome (Guindon et al., 2010).

### Nucleotide Sequence Accession Number

The complete genome sequence of *Bacillus* sp. DU-106 was deposited in the Genomes database under the accession number CP026607 (BioProject: PRJNA432450). The raw data of *Bacillus* sp. DU-106 genome sequencing data have been uploaded to the NCBI Sequence Read Archive as accession number SRP157864 (BioProject: PRJNA485855).

## Results and Discussion

### Identification and L-Lactic Acid Production Capacity of DU-106

**FIGURE 1:**
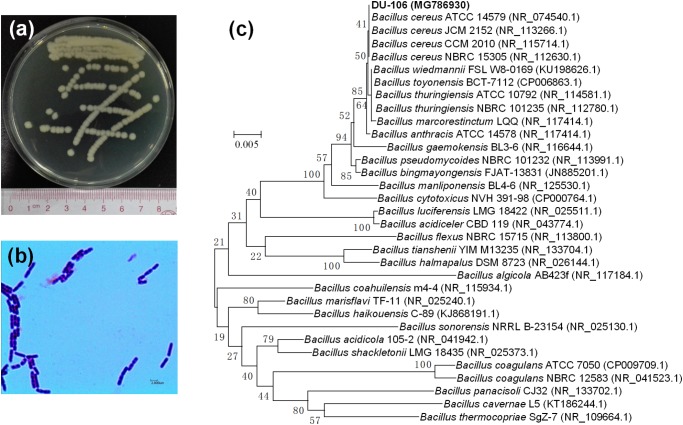
Colonial morphology **(a)**, Gram stain image **(b)**, and phylogenetic analysis **(c)** of strain DU-106. Strain DU-106 is grew on MRS plate after 48 h cultivation at 37°C. Neighbor-joining phylogenetic tree is based on the 16S rRNA gene sequences of strain DU-106 and representative strains from GenBank. Significance of each branch is indicated by a bootstrap value calculated for 1000 replicates. Numbers at branching points are bootstrap values >50%. GenBank accession numbers are given in parentheses. Bar, 0.005 substitutions per nucleotide position.

*Bacillus* sp. DU-106 was gram-stain-positive rod (**Figure 1b**). It was placed in the genus *Bacillus* by morphological observation and 16S rRNA gene sequencing (**Figure 1**). The phylogenetic tree indicated that the strain DU-106 formed a clade with the *Bacillus cereus* group (**Figure 1c**) with 16S rRNA gene sequence similarity levels of 97.05%-99.92%, whereas their similarity to other *Bacillus* species was below 95.7%. It is well known that these species of *B. cereus* group are genetically very close (Guinebretière et al., 2013).

**FIGURE 2:**
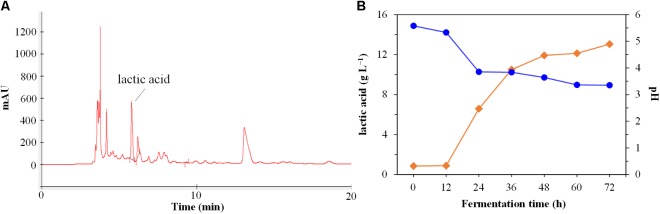
High performance liquid chromatography (HPLC) chromatograms of fermentation culture **(A)** of strain DU-106 after 48 h fermentation at 37°C in 200 mL MRS broth. Time course of lactic acid production and pH **(B)** during the fermentation by DU-106 in MRS medium at 37°C. The pH was declined from initial 5.58 to 3.35 after 72 h fermentation at 37°C.

As shown in **Figure 2**, the strain DU-106 could ferment glucose to L-lactic acid (**Figure 2A**), and the production of L-lactic acid was reached 13.04 g L^−1^ in MRS medium (Difco Laboratories, Detroit, MI, United States) after 48 h fermentation at 37°C (**Figure 2B**). Accordingly, the pH value was declined from initial 5.58 to 3.35 after 72 h fermentation at 37°C. The strain DU-106, to the best of our knowledge, is the first effective L-lactic acid producer of *B. cereus* group.

### *In vitro* Determination of Probiotic Characteristics

**FIGURE 3:**
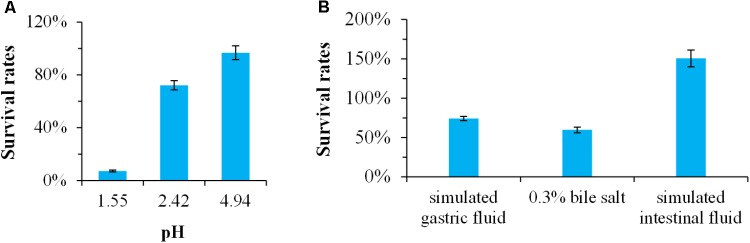
The potential probiotic properties of *Bacillus* sp. DU-106 *in vitro*. Survival rates of *Bacillus* sp. DU-106 in different pH **(A)** and the simulated gastric fluid, 0.3% bile salt, and simulated intestinal fluid **(B)** after 2 h treatment.

Generally, probiotics must have the ability to survive passage through the stomach and small intestine (Tyagi and Prasad, 2015). Therefore, resistance to the low pH of the gastric juice in the stomach and the bile salt in the small intestine is a prerequisite for probiotic (Shehata et al., 2016). In this study, the survival rates of *Bacillus* sp. DU-106 in pH 1.55, 2.42, and 4.94 were 7.14, 72.09 and 96.77% (**Figure 3A**), respectively. The survival rates of *Bacillus* sp. DU-106 in simulated gastric fluid, 0.3% bile salt, and simulated intestinal fluid after 2 h treatment were 74.29, 59.67, and 150.52% (**Figure 3B**), respectively. These results indicated that strain DU-106 might possess excellent potential probiotic properties.

### *In vivo* Toxicological Evaluation of DU-106 With Laboratory Mice

**FIGURE 4:**
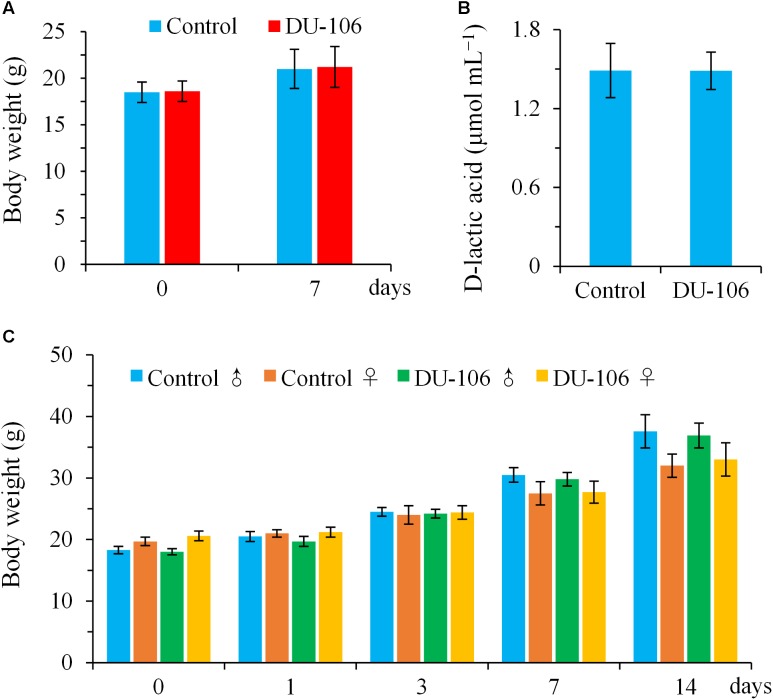
Toxicological evaluation of *Bacillus* sp. DU-106 *in vivo* with laboratory mice. Mean body weights **(A)** and D-lactic acid levels of blood **(B)** of BALB/c mice after continuous gavage for 7 days (3.0 × 10^8^ cfu d^−1^). Mean body weights of male and female KM mice during acute oral toxicity **(C)**.

No significant differences (*p* > 0.05) in body weights and D-lactic acid levels of blood were detected in BALB/c mice in comparison to the control groups after continuous gavage for 7 days (**Figures 4A,B**). There was no clinical abnormality found in any BALB/c animal during this study. All male and female KM mice survived, gained normal body weight, and appeared active and healthy during acute oral toxicity (**Figure 4C**). No gross abnormalities or pathological alterations were noted for any of the KM mice when necropsied at the end of the 14-day observation period. The acute oral LD_50_ of *Bacillus* sp. DU-106 was found to be greater than 1.0 × 10^12^ cfu kg^−1^ body weight. The results of acute oral toxicity test indicated that dose of 1.0 × 10^12^ cfu kg^−1^ body weight did not give rise to acute toxicity in KM mice, hence this dose was considered to be safe for humans (Yang et al., 2016).

### Genome Features of DU-106

**FIGURE 5:**
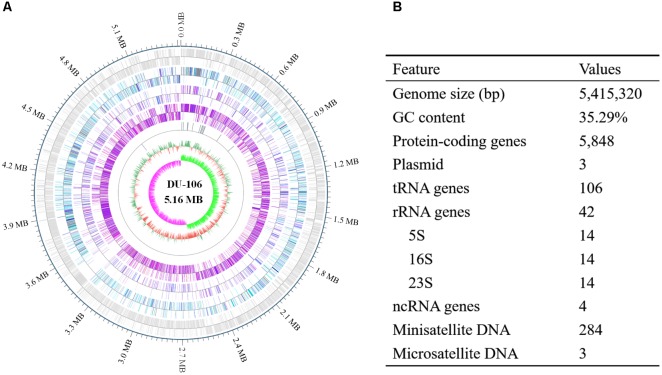
Circular representation **(A)** and features of the genome of *Bacillus* sp. DU-106 **(B)**. From the outer to inner circle: (1) predicted protein-coding sequences (CDSs); (2) predicted CDSs related to COG categories; (3) predicted CDSs related to KEGG categories; (4) predicted CDSs related to GO categories; (5) tRNA, rRNA and ncRNA distribution; (6) GC content; and (7) GC skew.

After filtering, approximately 1038 Mb Pacbio subreads and 1354 Mb HiSeq clean data were generated. The genome of strain DU-106 contained one 5,415,320 bp circular chromosome (**Figure 5A**) and three circular plasmids. The G + C contents of the chromosome and plasmids were 35.29, 31.82, 33.65, and 29.38%, respectively. A total of 5848 protein-coding genes (CDSs), 42 rRNA and 106 tRNA genes were predicted in chromosome sequence (**Figure 5B**). Among these CDSs, 3327 genes were classified into 20 clusters of orthologous groups (COG) functional categories (**Supplementary Table S1**). More than 35% genes were involved in these functions of amino acid transport and metabolism, transcription, carbohydrate transport and metabolism, energy production and conversion, and Inorganic ion transport and metabolism (**Supplementary Table S1**). According to the annotation, *Bacillus* sp. DU-106 was predicted to possess complete metabolic pathways, including glycolysis, the tricarboxylic acid cycle, and the pentose phosphate pathway.

### Comparative Genome Analyses

**FIGURE 6:**
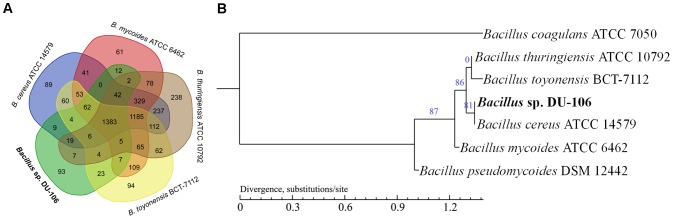
Comparative genome analyses of *Bacillus* sp. DU-106 and other *Bacillus* strains. Venn diagram of the genome comparison of *Bacillus* sp. DU-106 with other *B. cereus* strains **(A)** Venn diagram displays the orthologous genes between *B. cereus* ATCC 14579, *B. thuringiensis* ATCC 10792, *B. toyonensis* BCT-7112, and *B. mycoides* ATCC 6462. The maximum likelihood phylogenetic tree was constructed with PhyML based on SNP differences across the whole genome **(B)** Bootstrap support values were calculated from 100 replicates.

Chromosomes of the *Bacillus cereus* group exhibit a high level of synteny and protein similarity, with limited differences in gene content (Rasko et al., 2005). The strain DU-106 showed a high synteny of 92.39% with *B. cereus* ATCC 14579, 78.12% with *B. thuringiensis* ATCC 10792, 88.14% with *B. toyonensis* BCT-7112, 80.19% with *B. mycoides* ATCC 6462, and 64.59% with *B. pseudomycoides* DSM 12442, but displayed a low synteny of 4.82% with *B. coagulans* ATCC 7050 (**Supplementary Figure S1**). The over all core genome of these species is comprised of 1,383 orthologs with exception of *B. pseudomycoides* DSM 12442 and *B. coagulans* ATCC 7050 (**Figure 6A**). The genomes of strain DU-106 and *B. cereus* ATCC 14579 appear to be most similar by sharing 1,525 orthologs, whereas 1,489 orthologs were found between strain DU-106 and *B. toyonensis* BCT-7112. A total of 93 unique coding sequences in the genome of strain DU-106 were identified in the comparison (**Figure 6A**). In addition, a maximum likelihood phylogenetic tree based on SNP differences across the whole genome revealed that *Bacillus* sp. DU-106 shown the highest similarity with *B. cereus* ATCC 14579 (**Figure 6B**).

### Genomic Characterization of Probiotic Potency

Furthermore, we performed genomic data integrating with phenotypic assays to have a comprehensive view of relevant probiotic potency and safety aspects for the strain DU-106. *Bacillus* sp. DU-106 carried genes known to be involved in lactate synthesis including L-lactate dehydrogenase, D-lactate dehydrogenase, lactaldehyde dehydrogenase, and malate/lactate dehydrogenase. The genes coding for L-lactate dehydrogenase were also found in *B. cereus* ATCC 14579 and *B. coagulans* ATCC 7050 (Mols and Abee, 2011; Johnson et al., 2015). Moreover, genes encoding chaperone proteins DnaK, DnaJ and ClpB, ectoine biosynthesis, fatty acid synthesis, and ABC transporters for glycine betaine uptake were also identified in the genome sequence (**Table 1**). These proteins provide resistance to heat shock, acid and oxidative stress across all the probiotic genomes (Dopson et al., 2016). Interestingly, for examples, the gene involved in acid resistance of glycine betaine ABC transport system and fatty acid synthesis were found in *B. coagulans* but not *B. cereus* (Mols and Abee, 2011; Johnson et al., 2015). Betaine could enhance lactic acid production of *B. coagulans* by protecting L-lactate dehydrogenase activity and cell growth under osmotic inhibition (Xu and Xu, 2014). Changes in membrane fatty acid composition and content affected membrane fluidity, which also involved in the adaptation of acid stress (Wang et al., 2005). Previous work indicated that controlling the intracellular concentrations of fatty acids by acting on environmental conditions was an interesting way to improve the cryotolerance of *Lactobacillus bulgaricus* CFL1 (Streit et al., 2008). The genes involved in fatty acid synthesis were detected in the *Bacillus* sp. DU-106 (**Table 1**), as well as in the *B. coagulans*, but not found in *B. cereus* (Mols and Abee, 2011; Johnson et al., 2015).

**Table 1:** Predicted genes involved in probiotic potency in *Bacillus* sp. DU-106.

Predicted function	Predicted genes		
	*Bacillus* sp. DU-106	*B. cereus* 14579	*B. coagulans* 7050
**Lactate synthesis**	*lldh*, *dldh*, *ldh*, *m*/*ldh*	*lldh*	*lldh*
**Virulence gene**	*hlyIII*, *hlyBL*	*hlyIII*, *hlyBL*, *cytK*, *nheA*	*hlyIII*
**Adhesion protein**	*cbp*, *fbpIII*	*cbp*, *fbp*	*fbpA*
**Acid resistance-related**			
Protection or repair of macromolecules	*dnaK*, *dnaJ*, *clpB*, *groES*, *groEL*, *recA*, *uvrABC*	*dnaK*, *dnaJ*, *clpB*, *groES*, *recA*, *uvrC*	*dnaK*, *dnaJ*, *clpB*, *groEL*, *recA*, *uvrABC*
Fatty acid synthesis	*fabF*, *fadD*, *fabH*, *fabI*, *accC*	–	*fabF*, *fadD*, *fabH*, *fabI*, *accC*
Alkali production	*arcA*, *arcC*	*arcA*, *arcC*	–
Transcriptional regulators	*sigB*, *ctsR*, *hrcA*, *crp* family	*sigB*, *ctsR*, *hrcA*, *crp* family	*sigB*, *ctsR*, *hrcA*, *crp* family
Acid shock response	*aspS*	–	*aspS*
Metabolic rearrangements	*alsD*, *csa*	*alsD*	*alsD*
Glycine betaine ABC transport system	*opuAB*	–	*opuCC*
**Bile salt resistance**	*bass*, *cgh*	*bass*, *cgh*	*bass*

*cbp, gene for a potential collagen-binding protein; fbpIII, gene for fibronectin-binding protein type III domain; bass, gene for bile acid sodium symporter; cgh, gene for choloylglycine hydrolase; and –, not found.*

We also identified one gene coding for bile acid sodium symporter and two genes coding for choloylglycine hydrolase that provides the organism resistance to bile salt (Indu et al., 2016). Moreover, we annotated genes coding for adhesion proteins including collagen-binding protein and fibronectin-binding protein type III domain, those potentially aiding in bind to the digestive tract and reduce pathogenic adherence in probiotics (Granato et al., 2004). These findings at the genomic level further agreed that *Bacillus* sp. DU-106 possessed potential probiotic properties (**Figure 3**).

Generally, many pathogenic and virulence associated genes were predicted in the genome of *B. cereus* group food-poisoning strains (Arnesen et al., 2008; Rossi et al., 2018). For examples, virulence gene of *cytK* coding for cytotoxin K was commonly spread in *B. cereus* and *B. thuringiensis*. In this study, strain DU-106 genome lacks the genes encodes cereulide synthetase, enterotoxin FM, and cytotoxin K. These findings at the genomic level are in agreement with the proven safety of acute oral toxicity test (**Figure 4**). However, several hemolysin family genes were found in the genome of strain DU-106 (**Table 1**). We speculated that the hemolysin was inactive protein, since some of their toxin components show important amino acid changes in presence of the Sec type signal peptide (Fagerlund et al., 2010; Jiménez et al., 2013). This result was consist with previously reports, which reported that *B. toyonensis* BCT-7112^T^, a member of *B. cereus* group, contained the virulence gene of non-hemolytic enterotoxin an hemolysin BL, but had been used as a probiotic in animal feed in Japan over 40 years (Jiménez et al., 2013). Similarly, the probiotic *B. coagulans* ATCC 7050 also contained the gene for hemolysin (Johnson et al., 2015). Overall, these results indicated that *Bacillus* sp. DU-106 is prospective and potential probiotic candidates for industrial applications subject to further detailed investigations on their suitability for consumption as probiotic.

## Author Contributions

PL and BD conceived and designed the experiments. PL and WT performed the experiments. PL, ZJ, and ZL generated and analyzed the data. PL wrote the paper. XW analyzed the genomic data.

## Conflict of Interest Statement

The authors declare that the research was conducted in the absence of any commercial or financial relationships that could be construed as a potential conflict of interest.

## Supplementary Material

TABLE S1 COG functional categories of the complete genome sequence of *Bacillus* sp. DU-106.

FIGURE S1 Genomic overview of the similarity between the complete genome of strain DU-106 *Bacillus cereus* ATCC 14579, *Bacillus thuringiensis* ATCC 10792, *Bacillus toyonensis* BCT-7112, *Bacillus mycoides* ATCC 6462, *Bacillus pseudomycoides* DSM 12442, and *Bacillus coagulans* ATCC 7050. The rainbow color lines shows extensive synteny between genomes. The gaps between the blocks show differences in genomic content between genomes. Similarity comparison between complete genomes was performed with MUMmer 3.23 (http://mummer.sourceforge.net/) and LASTZ 1.03.54 (http://www.bx.psu.edu/miller_lab/dist/README.lastz-1.02.00/).
