# Supplementary material for: Genomic Characterization and Probiotic Potency of Bacillus sp. DU-106, a Highly Effective Producer of L-Lactic Acid Isolated From Fermented Yogurt
Source: Front Microbiol. 2018 Sep 20;9:2216. doi: 10.3389/fmicb.2018.02216 (PMC6158304; doi:10.3389/fmicb.2018.02216)
Supplement: TABLE S1 — COG functional categories of the complete genome sequence of Bacillus sp. DU-106. [file Table_1.DOCX]

**Table S1** COG functional categories of the complete genome sequence of *Bacillus* sp. DU-106.

| COG code | Functional Category | DU-106 |
| --- | --- | --- |
| B | Chromatin structure and dynamics | 1 |
| C | Energy production and conversion | 187 |
| D | Cell cycle control, cell division, chromosome partitioning | 30 |
| E | Amino acid transport and metabolism | 361 |
| F | Nucleotide transport and metabolism | 94 |
| G | Carbohydrate transport and metabolism | 211 |
| H | Coenzyme transport and metabolism | 136 |
| I | Lipid transport and metabolism | 108 |
| J | Translation, ribosomal structure and biogenesis | 182 |
| K | Transcription | 296 |
| L | Replication, recombination and repair | 137 |
| M | Cell wall/membrane/envelope biogenesis | 148 |
| N | Cell motility | 35 |
| O | Posttranslational modification, protein turnover, chaperones | 92 |
| P | Inorganic ion transport and metabolism | 223 |
| Q | Secondary metabolites biosynthesis, transport and catabolism | 83 |
| R | General function prediction only | 456 |
| S | Function unknown | 286 |
| T | Signal transduction mechanisms | 139 |
| U | Intracellular trafficking, secretion, and vesicular transport | 39 |
| V | Defense mechanisms | 82 |
| W | Extracellular structures | 1 |
| Total |  | 3327 |
