# Supplementary figures and images for: Genomic Characterization and Probiotic Potency of Bacillus sp. DU-106, a Highly Effective Producer of L-Lactic Acid Isolated From Fermented Yogurt
Source: Front Microbiol. 2018 Sep 20;9:2216. doi: 10.3389/fmicb.2018.02216 (PMC6158304; doi:10.3389/fmicb.2018.02216)

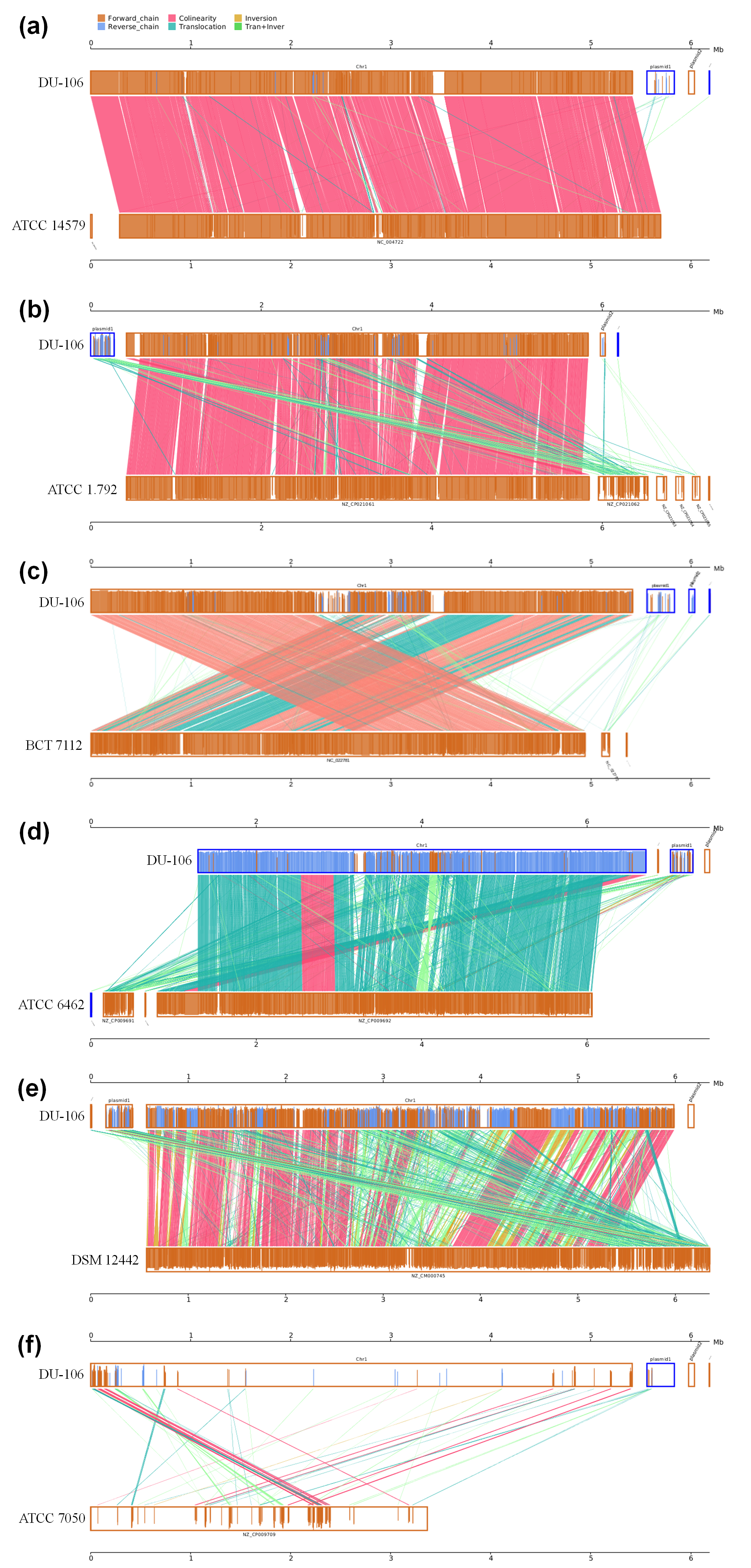

Supplement: FIGURE S1 — Genomic overview of the similarity between the complete genome of strain DU-106 Bacillus cereus ATCC 14579, Bacillus thuringiensis ATCC 10792, Bacillus toyonensis BCT-7112, Bacillus mycoides ATCC 6462, Bacillus pseudomycoides DSM 12442, and Bacillus coagulans ATCC 7050. The rainbow color lines shows extensive synteny between genomes. The gaps between the blocks show differences in genomic content between genomes. Similarity comparison between complete genomes was performed with MUMmer 3.23 (http://mummer.sourceforge.net/) and LASTZ 1.03.54 (http://www.bx.psu.edu/miller_lab/dist/README.lastz-1.02.00/). [file Image_1.TIF]
